# Obesity Increases the Severity and Mortality of Influenza and COVID-19: A Systematic Review and Meta-Analysis

**DOI:** 10.3389/fendo.2020.595109

**Published:** 2020-12-21

**Authors:** Xue Zhao, Xiaokun Gang, Guangyu He, Zhuo Li, You Lv, Qing Han, Guixia Wang

**Affiliations:** ^1^Department of Endocrinology and Metabolism, The First Hospital of Jilin University, Changchun, China; ^2^Department of Orthopedics, The Second Hospital of Jilin University, Changchun, China

**Keywords:** obesity, COVID-19, influenza, mortality, severe outcome

## Abstract

Since December 2019, COVID-19 has aroused global attention. Studies show the link between obesity and severe outcome of influenza and COVID-19. Thus, we aimed to compare the impacts of obesity on the severity and mortality of influenza and COVID-19 by performing a meta-analysis. A systematic search was performed in MEDLINE, EMASE, ClinicalTrials.gov, and Web of Science from January 2009 to July 2020. The protocol was registered onto PROSPERO (CRD42020201461). After selection, 46 studies were included in this meta-analysis. The pooled odds ratios (ORs) with 95% confidence intervals (CIs) were analyzed. We found obesity was a risk factor for the severity and mortality of influenza (ORsevere outcome = 1.56, CI: 1.28-1.90; ORmortality = 1.99, CI: 1.15-3.46). For COVID-19, obesity was a significant risk factor only for severe outcome (OR = 2.07, CI: 1.53-2.81) but not for mortality (OR = 1.57, CI: 0.85-2.90). Compared with obesity, morbid obesity was linked with a higher risk for the severity and mortality of both influenza (OR = 1.40, CI: 1.10-1.79) and COVID-19 (OR = 3.76, CI: 2.67-5.28). Thus, obesity should be recommended as a risk factor for the prognosis assessment of COVID-19. Special monitoring and earlier treatment should be implemented in patients with obesity and COVID-19.

## Introduction

Since December 2019, countries globally have been suffering from the spread of COVID-19, which is also known as SARS-CoV-2 (1, 2). The latest information indicates that there are more than 48 million COVID-19 cases around the world, according to the data on November 6, 2020. Based on the 1918 “Spanish” influenza pandemic and 2009 influenza A (H1N1) pandemic (3), studies show the close relationship between obesity and virus infection as well as mortality (4, 5). Significantly, special attention should be paid to obese patients. Due to the prolonged pandemic of COVID-19, tons of expenses have been spent on medical fields, which has influenced the social economy extremely (6). Thus, investigating the influencing factors and susceptible population are the most important things to prevent the pandemic of COVID-19. Evidence from previous influenza studies might provide referential and warning values for the understanding and control of COVID-19.

Obesity is one of the most important diseases affecting people’s health with dramatically increased morbidity year by year (7, 8). Studies indicate that obesity is linked with increased influenza infection. Because of the disturbed immune regulation and metabolic homeostasis, patients with obesity present higher risks for the severe outcome and mortality of influenza (9, 10). Similarly, the latest studies show that COVID-19 patients with obesity are more likely to be admitted to the intensive care unit (ICU), be on mechanical support, and have a severe outcome (11, 12). Although some reviews and meta-analyses have been published on the topic of obesity and severe outcome of COVID-19, the data are updated every minute (13). An available and timely meta-analysis is urgent. Moreover, the differences and comparisons of severity, outcome, and mortality between influenza and COVID-19 in patients with obesity are unknown so far. Thus, to compare the effect of obesity on influenza and COVID-19 is of great value and importance for us to learn about COVID-19.

In the present study, we aimed to systematically review and compare the effects of obesity on the infection, hospitalization, disease severity, and mortality of both influenza and COVID-19 based on the available evidence. In addition, we updated the meta-analysis by including the latest studies from other countries. We hope this meta-analysis provides more valuable information for the management of the COVID-19 pandemic.

## Methods

### Literature Search Strategy

A systematic search was conducted to identify papers available on MEDLINE, EMASE, ClinicalTrials.gov, and Web of Science for relevant studies from January 2009 to July 2020. The review protocol was registered onto PROSPERO (CRD42020201461). We developed a search strategy for MEDLINE based on medical subject heading (MeSH^®^) terms and text of target papers. Different possible variations and combinations of the following search terms were used: influenza, influenza A, influenza B, H1N1, H7N9, COVID-19, SARS-CoV-2, coronavirus, 2019 nCoV, obesity, obese, BMI, severity, outcome, and mortality. We also reviewed the reference lists of all included papers and relevant review papers to identify studies that the database searches might have missed. To minimize selection bias, two persons completed this work independently. Disagreements were resolved by consensus and by a third person. In the initial search, no filter for language preference was used. The literature search process is based on the PRISMA form (14).

### Inclusion and Exclusion Criteria

Articles were included or excluded on the basis of full-text articles. The following inclusion criteria were applied: 1) For the diagnosis of obesity, BMI was applied for classification according to the American Endocrine Society Scientific Statement on obesity management (15): ①normal weight: 18.5 kg/m^2^ < BMI < 25 kg/m^2^; ②overweight: 25 kg/m^2^ ≤ BMI < 30 kg/m^2^; ③obesity: BMI ≥ 30 kg/m^2^; ④morbid obesity: BMI ≥ 35 kg/m^2^. 2) COVID-19 infection is diagnosed according to the criteria established by the China National Health Commission (16) based on laboratory examinations, such as nasopharyngeal and oropharyngeal swab tests. Detection tests for coronavirus include reverse-transcription polymerase chain reaction (RT-PCR), real-time RT-PCR (rRT-PCR), and reverse transcription loop-mediated isothermal amplification (RT-LAMP). To identify patients earlier, two one-step quantitative RT-PCR (qRT-PCR) assays were developed to detect two different regions (ORF1b and N) of the SARS-CoV-2 genome. For patients suffering from fever, sore throat, fatigue, coughing, or dyspnea coupled with recent exposure, COVID-19 infection should be diagnosed with typical chest computerized tomography (CT) characteristics despite negative RT-PCR results. 3) Influenza infection is diagnosed with clinical manifestations and laboratory tests (17). The RT-PCR test is the most traditional yet powerful approach for identification of influenza viruses in most diagnostic labs around the world. Rapid influenza diagnostic tests are also applied by many countries, using monoclonal antibodies that target the viral nucleoprotein and employ either an enzyme immunoassay or immunochromatographic techniques. 4) Included studies should present data that could be extracted straightforwardly, and detailed characteristics on the population and studies should be provided. 5) Severe outcome of influenza and COVID-19 refers to admission to the ICU, requiring mechanical support, hypoxia requiring oxygen therapy, and increased mortality and death. 6) Only papers published in English were included in the present study, but abstracts of non-English papers were also reviewed to prevent missing information.

Studies were excluded if subjects had diabetes or other fatal chronic diseases or medication history. Fatal chronic disease refers to disease that could induce the severity of COVID-19 or influenza besides obesity, which might make it difficult to discuss the role of obesity on the risk and outcome of COVID-19 or influenza. For instance, fatal chronic diseases include cancer, severe autoimmune disease, chronic kidney disease, diabetes, cirrhosis, liver failure, and so on. As for medication history, it does not mean all kinds of treatment are excluded. Only medications that could cause interference in the outcome or mortality of COVID-19 or influenza were excluded. Case reports, reviews, meta-analyses, conference abstracts, and letters to the editor were also excluded.

### Data Extraction and Analysis

Both investigators (XZ and QH) initially screened all relevant titles and abstracts to confirm if the studies were related to the topics of present study. After the first round of reviewing, full-text articles were read by two persons. Data were extracted from studies meeting our inclusion criteria according to our self-made forms. Data on population characteristics, group descriptions, and odds ratio or relative risk (OR/RR) values were extracted. One investigator performed the data extraction (XZ), which was verified by a second investigator (QH). Data from influenza and COVID-19 studies were analyzed through meta-analysis guidelines. For influenza studies, we aimed to analyze the data on the influenza infection, hospitalization, disease severity, and mortality in patients with obesity or morbid obesity. For COVID-19 studies, we aimed to analyze the data on the disease severity and mortality in patients with obesity or morbid obesity. OR/RR and 95% confidence interval (95% CI) were presented as effect size for case control or cohort studies. Before data synthesis, heterogeneity was estimated using the Q test and I^2^ statistic. When *P* < 0.1 or I^2^>50%, indicating the existence of possible heterogeneity among studies, the random-effect model was applied. Sensitivity analyses were performed to explore the resources of heterogeneity in instances in which sufficient data were collected. The Begg’s test was used to evaluate publication biases. All analyses were completed with the software STATA 15.1 (StataCorp, College Station, TX, USA). All statistical tests were 2-tailed.

### Methodological Quality Assessment

The 9-star Newcastle-Ottawa Scale (NOS) was applied to assess the quality of the studies (18). The methodologies of studies that achieved 6/9 or more were classified as high quality, whereas those that scored less than 5/9 were classified as low quality.

## Results

### Literature Search and Study Characteristics

**Figure 1:**
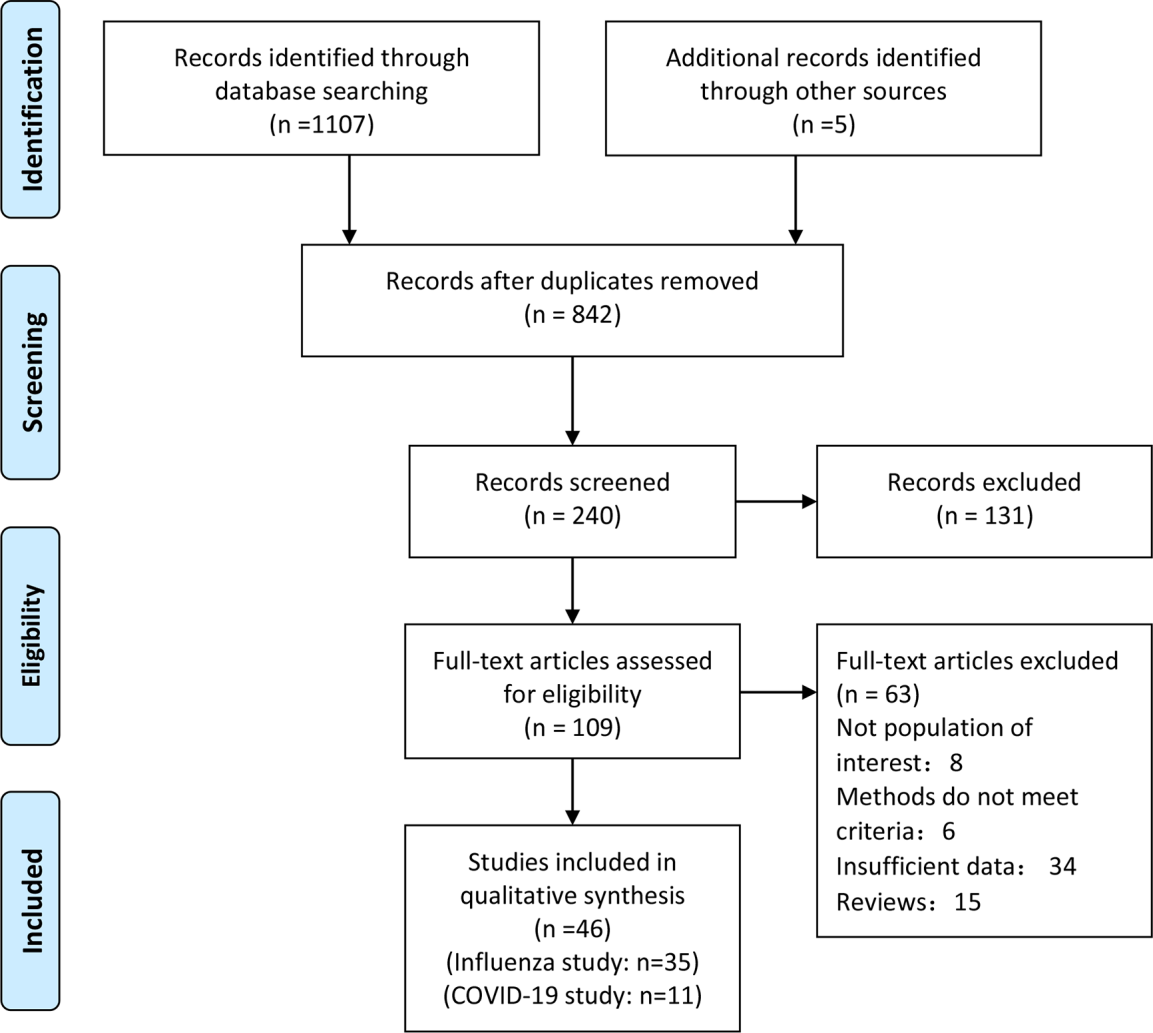
Flowchart on the literature selection process in this meta-analysis.

After careful selection, a total of 46 studies were included in the present meta-analysis; 11/46 studies were COVID-19 studies (11, 19–28), and 35/46 studies were influenza studies (29–61). A total of 4,023,895 patients were included for the studies on influenza, and 9,787 patients were included in the COVID-19 studies. The procedure of literature selection based on the PRISMA statement is shown in **Figure 1**. Details about the characteristics of the included studies are shown in **Tables 1** and **2**. The included studies were published between 2009 and 2020. Of the included COVID-19 studies, 4 were conducted in the United States, 3 were from China, and 1 each was from France, Singapore, Italy, and Mexico, respectively. Of the included influenza studies, 12 were conducted in the United States, 4 were from Spain, 6 were from China, 3 were from Canada, 2 were from UK, and 1 each was from the following countries: Mexico, Serbia, France, Iran, Romania, France, Brazil, Australia, and South Africa. The studies varied in sample size from 48 to 3,076,699. In addition, 41/46 studies enrolled both males and females, and 5 did not show the gender characteristic. Study quality assessment according to NOS is shown in **Tables 1** and **2**.

**Table 1:** Characteristics of included studies on the infection, hospitalization, severity, and mortality of influenza.

Author	Country	Year	Number	Women (%)	Age	Groups	Disease outcome	OR or RR (95% CI)	NOS
Ren et al. (29)	China	2013	686	48.40%	5-60	Overweight; Obesity	Severe influenza manifestation	Overweight: 3.70(2.04, 6.72) Obesity: 35.61 (7.96, 159.21)	7
Maier et al. (30)	US	2018	1783	74%	0-92	Obesity	Influenza	2.04 (1.35, 3.09)	6
Segaloff et al. (31)	US	2017	48	23%	21-91	Obesity Morbid obesity	Severe influenza outcomes	Obesity: ICU: 2.9 (0.5, 20.9); LRD: 2.1 (0.5, 8.6) Morbid obesity: ICU: 7.9 (0.9, 87.1); LRD: 4.0 (0.6, 35.0)	6
Moser et al. (32)	Mexico	2018	3248	64.25%	41.2 ± 16.2	Underweight; Obesity; morbid obesity	hospitalization	Underweight: 5.20 (1.67, 16.01) Obesity: 3.18 (1.73, 5.91) Morbid obesity: 18.4 (7.83, 47.4)	5
Neidich et al. (33)	US	2017	1022	64.68%	54 ± 15	Obesity	Influenza risk	Obesity: 2.01 (1.12, 3.06)	5
Murphy et al. (34)	UK	2016	6984	53.94%	35-63	Obesity	Influenza-like illness	Obesity: 1.16 (0.97, 1.38)	6
Yang et al. (35)	China	2013	66820	65.75%	65+	Underweight; Overweight; Obesity; Morbid obesity	influenza-associated mortality	Underweight: 1.081 (1.013, 1.154) Overweight: 0.981 (0.936, 1.028) Obesity: 1.018 (0.980, 1.058) Morbid obesity: 1.062 (0.972, 1.162)	6
Cocoros et al. (36)	US	2014	8266	59.63%	48+	Overweight; Obesity	Influenza-like illness	Overweight: 1.54 (0.56, 4.27) Obesity: 1.90 (0.68, 5.27)	6
Dragana et al. (37)	Serbia	2017	777	48.26%	65+	Obesity	Admission to ICU	Obesity: 3.32 (1.36, 8.14)	6
Charland et al. (38)	US	2012	3076699	N/A	0-80+	Obesity	Hospitalization	Obesity: 1.12 (1.07, 1.17)	6
Braun et al. (39)	US	2015	9048	65.6%	20+	Underweight; Overweight; Obesity; Morbid obesity	Influenza severity (admission to ICU)	Underweight: 1.13 (0.86, 1.49) Overweight: 0.80 (0.70, 0.94) Obesity: 0.90 (0.76, 1.08) Morbid obesity: 0.91 (0.77, 1.09)	5
Coleman et al. (40)	US	2012	2623	65.42%	45 ± 17	Obesity; Morbid obesity	Influenza	Obesity: 0.95 (0.75, 1.20) Morbid obesity: 1.10 (0.8, 1.52)	6
Morgan et al. (41)	US	2010	594	N/A	N/A	Obesity; Morbid obesity	Hospitalization; Death	Obesity: hospitalization: 1.5 (0.8, 2.8); death: 4.9 (2.4, 9.9) Morbid obesity: hospitalization: 3.1 (1.5, 6.6); death: 7.6 (2.1, 27.9)	6
Halvorson et al. (42)	US	2018	2811	61%	22+	Overweight; Obesity; Morbid obesity	Hospitalization	Overweight: 0.8 (0.6, 1.0) Obesity: 0.7 (0.5, 1.0) Morbid obesity: 0.9 (0.6, 1.2)	6
Van Kerkhove et al. (62)	US	2015	11086	10.5%	28-40	obesity	Hospitalization severe outcomes	Obesity: 1.6 (1.2, 2.1)	7
Louie et al. (43)	US	2010	534	52%	46 (20, 92)	Morbid obesity	Death	Morbid obesity: 2.8 (1.4, 5.9)	6
Jime´nez-Garcı´a et al. (44)	Spain	2012	11499	54.2%	18+	Obesity	Death	Obesity: 1.88 (1.07, 3.92)	5
Díaz et al. (45)	Spain	2011	416	41.93%	43.1 ± 12.2	Obesity	Death	Obesity: 1.56 (0.95, 2.54)	6
Tempia et al. (46)	South Africa	2016	465	64.95%	5+	Obesity	Hospitalization	Obesity: 12.1 (1.6, 88.8)	6
Martin et al. (47)	US	2013	161	35.4%	40-46	Obesity	Hospitalization; death	Hospitalization: 2.93(1.50, 5.71) death: 5.33 (0.61, 46.71)	5
Bijani et al. (48)	Iran	2016	55	43.6%	25.7 ± 16.9	Obesity	Severe manifestation	Obesity: 0.89 (0.08, 10.43)	5
Cui et al. (49)	China	2010	68	N/A	41 (18, 66)	Obesity	Death	Obesity: 23.06 (0.95, 2.54)	7
Santa-Olalla Peralta et al. (63)	Spain	2010	3025	N/A	38 (0,94)	Obesity	Severe outcomes	Obesity: 2.01 (1.38, 2.94)	6
Gilca et al. (50)	Canada	2011	716	54.05%	5+	Obesity; Morbid obesity	Hospitalization; ICU or death	Obesity: hospitalization: 1.3 (0.8, 2.0); death: 1.0 (0.5, 2.3) Morbid obesity: hospitalization: 2.0 (0.6, 6.2); death: 0.4 (0.09, 2.0)	6
Lenzi et al. (51)	Brazil	2011	4740	53.31%	0-90	Obesity	Hospitalization	Obesity: 2.994 (1.638, 5.472)	5
Yu et al. (52)	China	2011	9966	44%	22 (11, 39)	Obesity	Severe outcomes	Obesity: 1.54 (1.35, 1.76)	6
Viasus et al. (53)	Spain	2010	585	48.7%	39 (16, 87)	Morbid obesity	Severe outcomes	Morbid obesity: 6.7 (2.25, 20.19)	6
Tang et al. (53)	China	2010	457	42.23%	25 (6, 42)	Overweight; Obesity	Severe outcomes	Overweight: 3.13 (1.83, 5.36) Obesity: 4.05 (1.72, 9.52)	6
Karki et al. (55)	Australia	2018	246494	46.99%	50+	Obesity; Morbid obesity	Influenza incidence; Hospitalization	Incidence: Obesity: 1.27 (1.10, 1.46) Morbid obesity: 1.69 (1.24, 2.29) Hospitalization: Obesity: 1.57 (1.22, 2.01); Morbid obesity: 4.81 (3.23, 7.17)	9
Zhou et al. (56)	China	2015	65841	66.14%	65+	Obesity	Influenza mortality	Obesity: 1.19 (1.01, 1.42)	8
Campitelli et al. (57)	Canada	2014	396581	48.92%	18-64	Overweight; Obesity; Morbid obesity	Acute respiratory infection for influenza	Overweight: 1.10 (1.07, 1.13) Obesity: 1.17 (1.13, 1.22) Morbid obesity: 1.19 (1.12, 1.25)	8
Kwong et al. (58)	Canada	2011	82545	50.49%	18+	Obesity; Morbid obesity	Hospitalization	Obesity: 1.45 (1.03, 2.05) Morbid obesity: 2.12 (1.45, 3.10)	9
Pițigoi et al. (59)	Romania	2019	345	55.9%	74 (68, 80)	Obesity	Hospitalization	Obesity: 2.1 (1.3, 3.4)	7
Guerrisi et al. (60)	France	2019	6992	61%	0-75	Underweight Overweight; Obesity	Influenza-like illness	Underweight:0.92(0.77,1.11) Overweight:1.18(1.08,1.29) Obesity:1.28(1.14,1.44)	8
Myles et al. (61)	UK	2012	1520	52.6%	26(9,44)	Obesity	Severe outcomes	Obesity:2.22(1.18,4.18)	7

**Table 2:** Characteristics of included studies on the severity and mortality of COVID-19.

Author	Country	Year	Number	Women (%)	Age	Group	Outcome	OR (95%CI)	NOS score
Simonnet et al (11).	France	2020	124	27%	60 (51, 70)	Obesity; Morbid obesity	Severe manifestation; ICU	Obesity:3.45(0.83,14.31) Morbid obesity: 7.36 (1.63, 33.14)	6
Cai et al (19).	China	2020	383	52.21%	48 (39, 54)	Overweight; Obesity	Severe manifestation; ICU	Overweight: 1.84 (0.99, 3.43) Obesity: 3.4 (1.4, 8.26)	7
Wu et al (20).	China	2020	280	46%	43.12 ± 19	Obesity	Severe influenza outcomes	Obesity: 1.3 (1.09, 1.54);	5
Lighter et al (21).	US	2020	3615	N/A	N/A	Obesity; morbid obesity	Severe manifestation; ICU	Obesity: 1.8 (1.2, 2.7) Morbid obesity: 3.6 (2.5, 5.3)	5
Ong et al (22).	Singapore	2020	182	60%	43 (27, 52)	Obesity	Severe manifestation; ICU	Severe manifestation: 6.32 (1.23, 32.34) ICU: 3.13 (0.57, 17.13)	5
Gao et al (23).	China	2020	150	37.3%	35-63	Obesity	Severe manifestation and ICU	Obesity: 3 (1.22, 7.38)	6
Pettie et al (24).	US	2020	238	52.5%	58.5 ± 17	Obesity	mortality	Obesity: 1.7 (1.1, 2.8)	6
Hajifathalian et al (24).	US	2020	770	39.2%	64 ± 16.7	Obesity	ICU and mortality	ICU: 1.76 (1.24, 2.48) mortality: 1.15 (0.62, 2.14)	7
Busetto et al (26).	Italy	2020	92	38.1%	70.5 ± 13.3	Obesity	Admission to ICU; death	ICU: 11.65 (3.88, 34.96) mortality: 0.27 (0.03, 2.05)	6
Palaiodimos et al (27).	US	2020	200	51%	64 (54, 73.5)	Morbid obesity	mortality	Morbid obesity: 3.78 (1.45, 9.83)	7
Denova-Gutiérrez et al (28).	Mexico	2020	3844	42%	45.4 ± 15.8	Obesity	Severe outcome; ICU	Obesity: 1.43 (1.11, 1.83)	6

### Obesity Increases the Risk of Influenza Infection and Hospitalization

**Figure 2:**
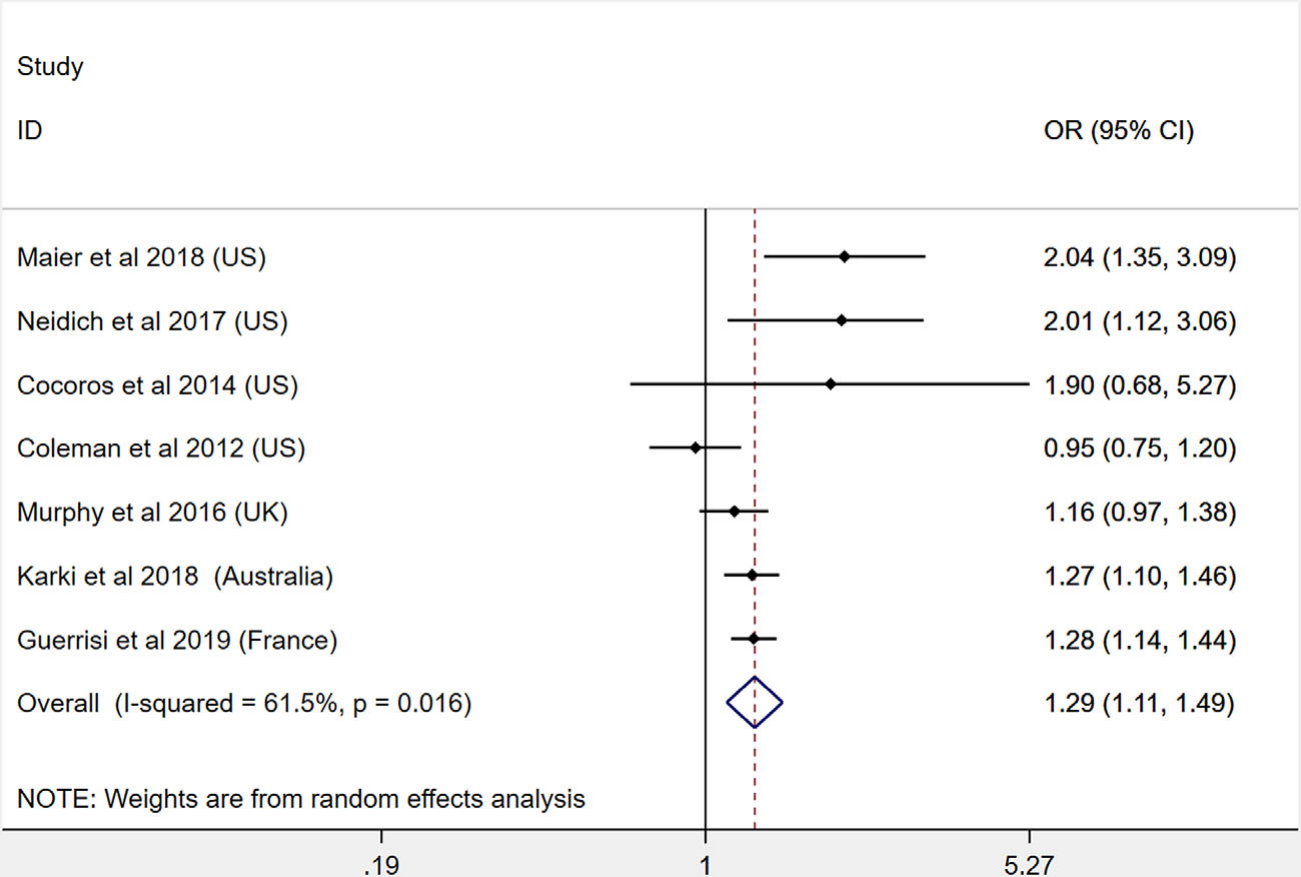
Odds ratios for influenza infection in patients with obesity versus patients without obesity.

A total of 7 studies were included for the meta-analysis of obesity and influenza infection risk (30, 33, 34, 36, 40, 55, 60). Our pooled analysis shows that patients with obesity had a significantly higher risk for influenza infection (OR: 1.29, 95% CI: 1.11-1.49, I^2^: 61.5%, *n*=7). This result is presented in **Figure 2**. Due to the heterogeneity between the included studies, we performed subgroup analysis according to different regions. The results show that the heterogeneity was decreased in non–North American countries (**Supplement Figure 1**).

**Figure 3:**
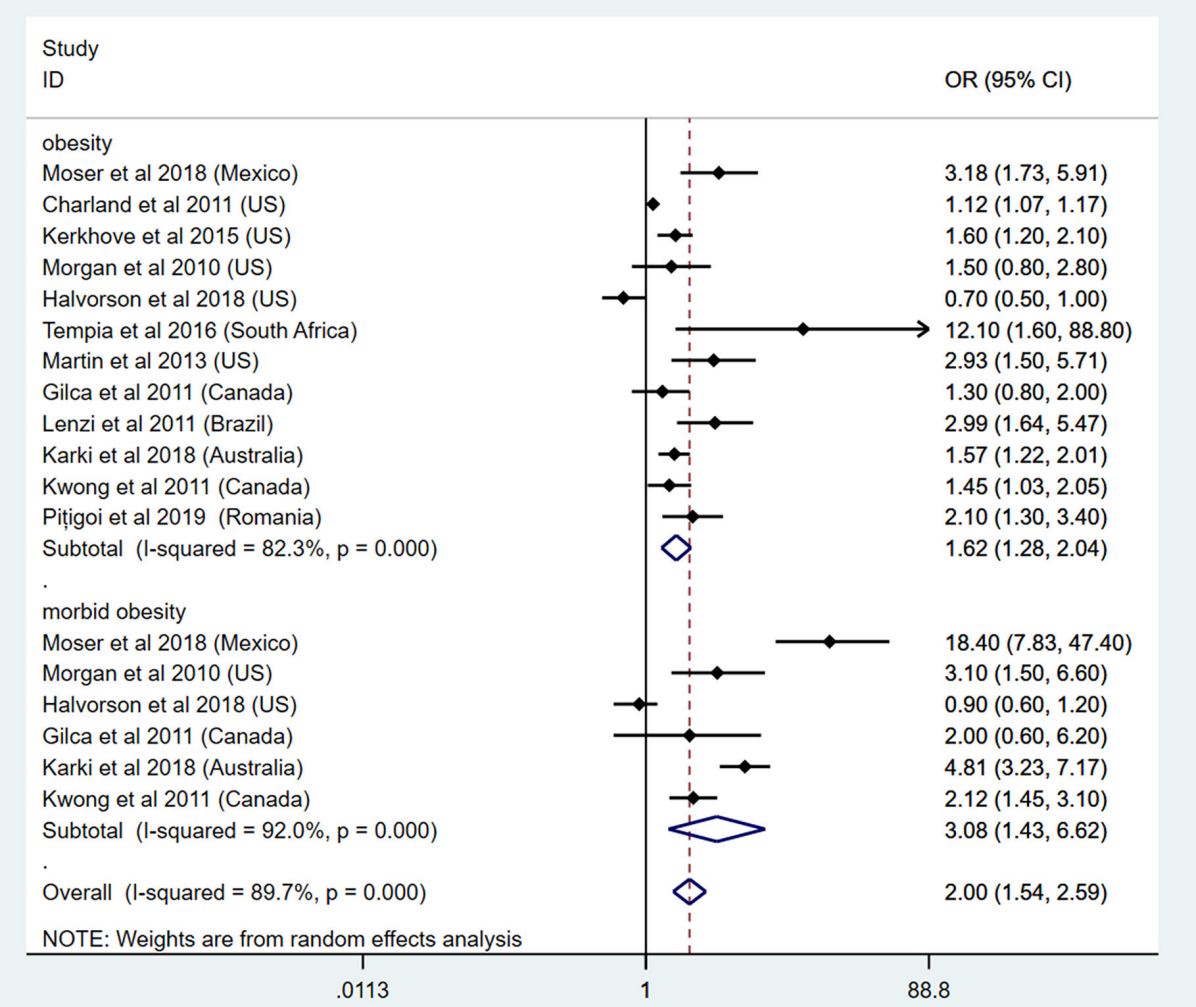
Odds ratios for influenza-induced hospitalization in patients with obesity and morbid obesity.

As for the risk of hospitalization, 18 studies were included in this part. To specify the effect of obesity classes on influenza hospitalization, 12 studies focused on the link between obesity and influenza hospitalization (32, 38, 41, 42, 46, 47, 50, 51, 55, 58, 59, 62). Among these, 6 studies focused on the link between morbid obesity and influenza hospitalization (32, 41, 42, 50, 55, 58). Our results show that patients with obesity had an increased risk for hospitalization because of influenza (OR: 1.62, 95% CI: 1.28-2.04, I^2^: 82.3%, *n*=12). In addition, patients with morbid obesity had extremely higher risk for hospitalization (OR: 3.08, 95% CI: 1.43-6.62, I^2^: 92%, *n*=6). The overall analysis showed consistent results (OR: 2.00, 95% CI: 1.54-2.59, I^2^: 89.7%, *n*=18). This result is presented in **Figure 3**. As for the subgroup analysis, the heterogeneity between the included studies did not show any decrease after subgroup analysis by different regions (**Supplement Figure 1**).

### Obesity Aggravates Disease Severity and Increases the Risk of ICU Admission in Both Influenza and COVID-19

**Figure 4:**
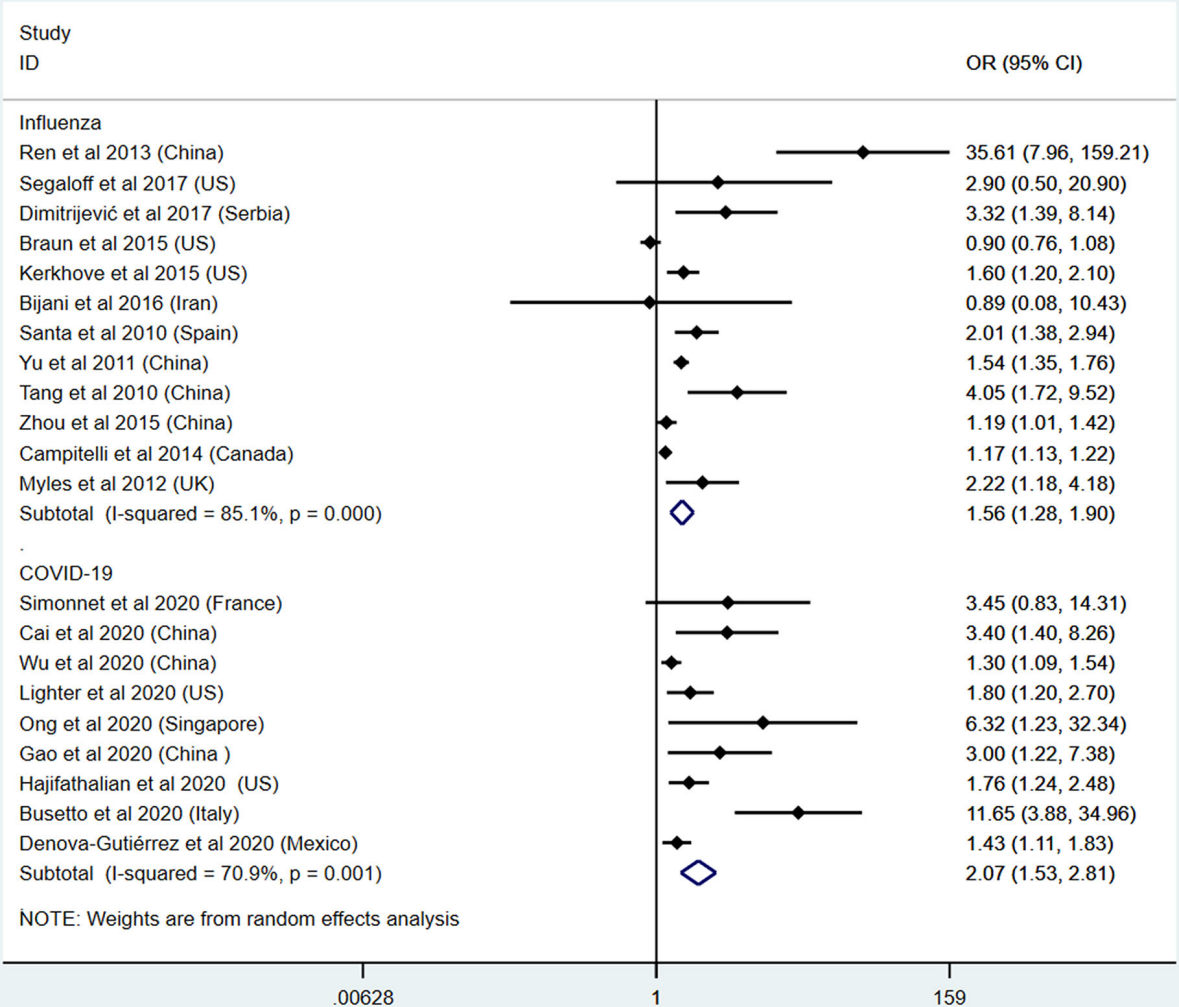
Odds ratios for severe outcome or admission to ICU in patients with obesity and influenza or COVID-19.

**Figure 5:**
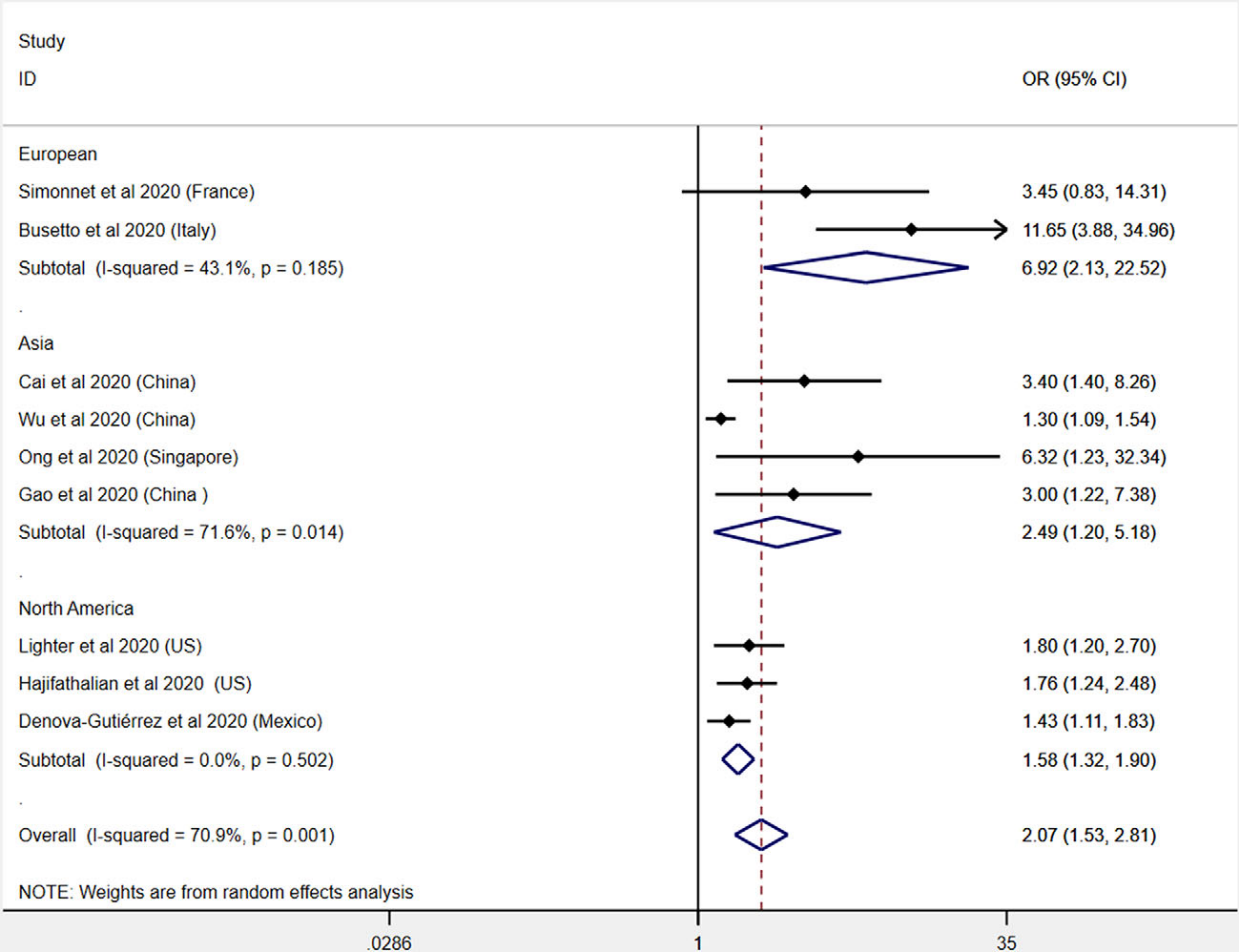
Subgroup analysis (by region) on the effects of obesity on the severity and mortality of COVID-19.

The severe outcomes consist of severe manifestations, admission to ICU, and requirement for mechanical supports. In this section, 12 influenza studies and 9 COVID-19 studies were included (11, 19–23, 25, 26, 28–31, 37, 39, 48, 52, 54, 56, 57, 61, 62). The pooled analysis results show that patients with obesity had an increased risk for severe outcome of both influenza (OR: 1.56, 95% CI: 1.28-1.90, I^2^: 85.1%, *n*=12) and COVID-19 (OR: 2.07, 95% CI: 1.53-2.81, I^2^: 70.9%, *n*=9). Compared with influenza, obesity patients with COVID-19 might present a higher risk for ICU admission and worse outcomes. This result is presented in **Figure 4**. To further explore the heterogeneity between the included studies, subgroup analysis was applied by different regions (**Supplement Figure 1**). The heterogeneity was significantly decreased in each subgroup of COVID-19 studies, indicating the region characteristic for the COVID-19 studies (**Figure 5**).

### Obesity Increases the Mortality of Influenza but Has No Adverse Effect on the Mortality of COVID-19

**Figure 6:**
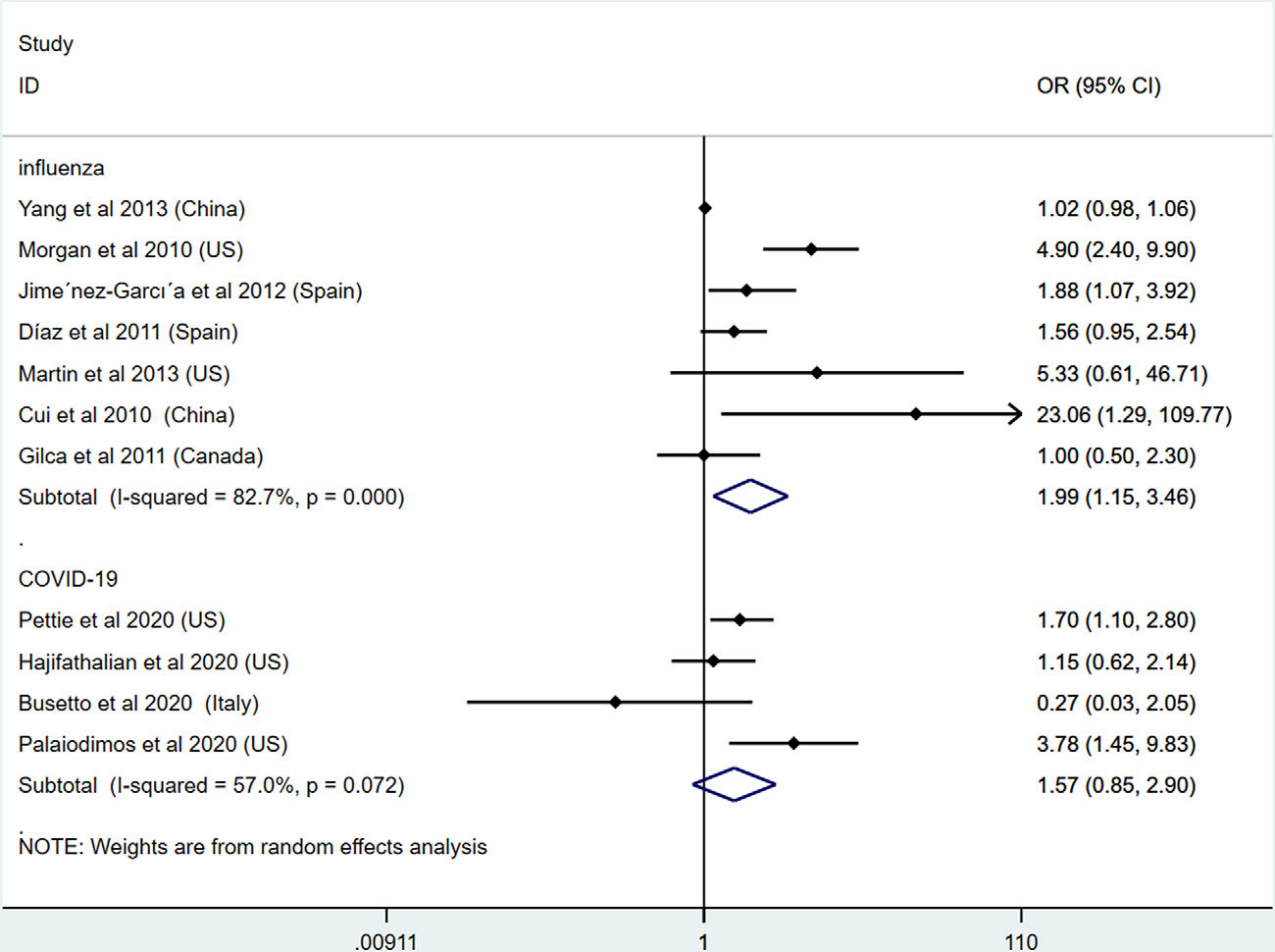
Odds ratios for the mortality of patients with obesity and influenza or COVID-19.

In addition to the severe outcome of both diseases, we then detected the effects of obesity on the mortality of influenza and COVID-19. In this section, 7 studies on influenza and 4 studies on COVID-19 were included (24–27, 35, 41, 44, 45, 47, 49, 50). Our results show that patients with obesity presented higher mortality of influenza (OR: 1.99, 95% CI: 1.15-3.46, I^2^: 82.7%, *n*=7). However, inconsistent with influenza studies, our results show no link between obesity and mortality of COVID-19 (OR: 1.57, 95% CI: 0.85-2.90, I^2^: 57%, *n*=12). This result demonstrates that, although obesity could aggravate the severe outcome of COVID-19, obesity does not increase the death rate based on available studies. This result is presented in **Figure 6**. To further explore the heterogeneity of the included studies, subgroup analysis was applied by different regions (**Supplement Figure 1**).

### Morbid Obesity Is Linked to Severity and Mortality of Both Influenza and COVID-19

**Figure 7:**
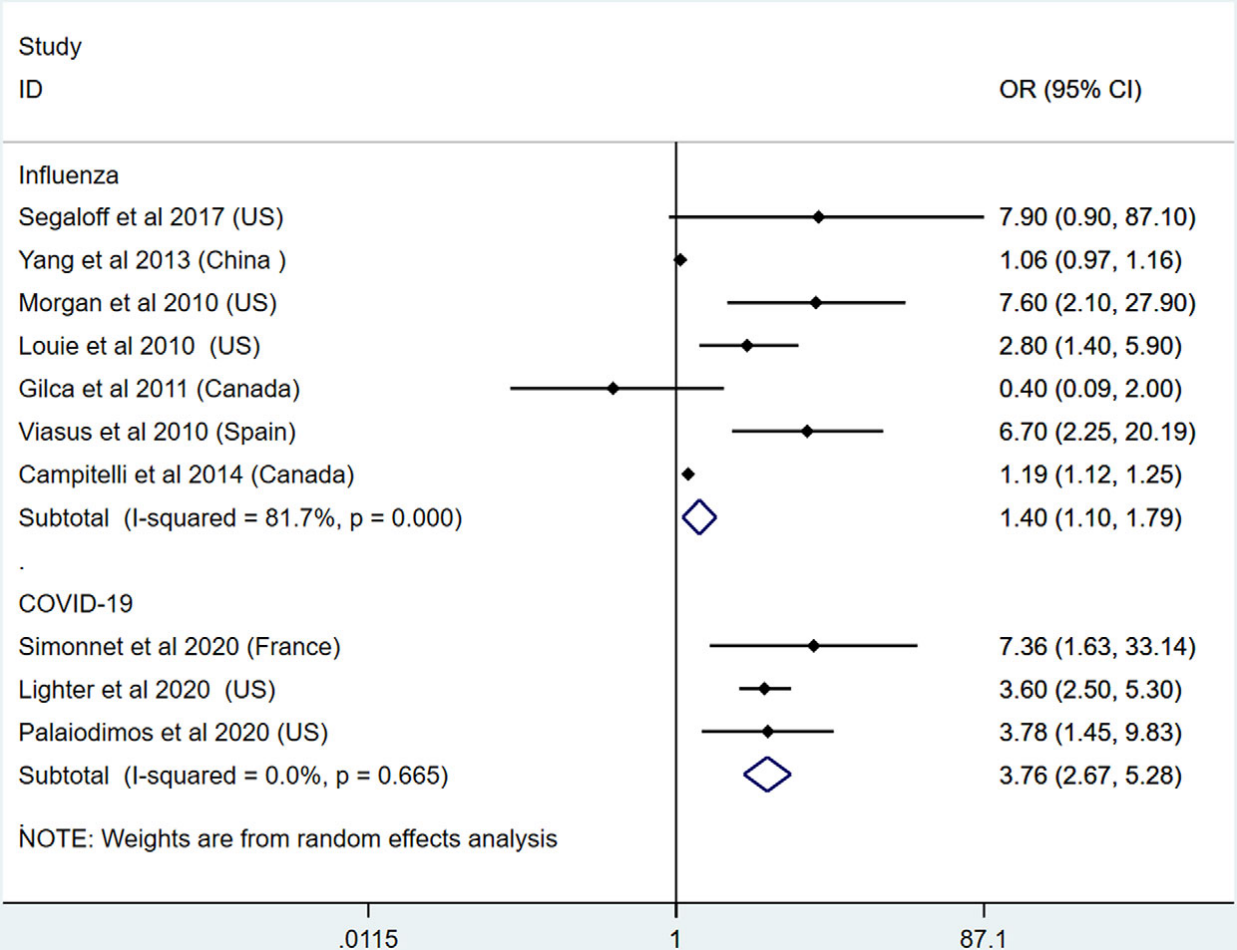
Odds ratios for severe outcome and mortality in patients with morbid obesity and influenza or COVID-19.

To detect the effects of different obesity classes on the severity and mortality of influenza and COVID-19, we applied the meta-analysis on morbid obesity studies. In this section, 7 studies on influenza and 3 studies on COVID-19 were included (11, 21, 27, 31, 35, 41, 43, 50, 53, 57). The results show that morbid obesity is a significant predictor or risk factor for the severe outcomes and death of influenza (OR: 1.40, 95% CI: 1.10-1.79, I^2^: 81.7%, *n*=7) and COVID-19 (OR: 3.76, 95% CI: 2.67-5.28, I^2^: 0%, *n*=3). Compared with influenza, COVID-19 infection in patients with morbid obesity induced higher mortality. This result is presented in **Figure 7**. Due to the limited number of studies, subgroup analysis was not performed.

### Publication Bias Assessment and Sensitivity Assessment

Begg’s tests were applied to evaluate publication bias. Because of the limited number of studies included in each section, no conclusive information was found after Begg’s tests and sensitivity assessment. The results are shown in **Supplement Figure 1**.

## Discussion

### The Influence of Obesity on the Severity and Mortality of Influenza and COVID-19

In the present meta-analysis, we systematically reviewed studies focusing on the influence of obesity on the severity and mortality of influenza and COVID-19. The results show obesity could increase the risk and severe outcomes of both influenza and COVID-19. Higher mortality of influenza was found in patients with obesity. Compared with influenza, COVID-19 patients with obesity present higher risk of severe outcomes and admission to ICU but not mortality. These results indicate the characteristic of COVID-19, which is higher infection rate and lower mortality. Thus, more strategies on weight control and physical regulation should be encouraged to decrease the risk of COVID-19 infection and its development.

The impact of different obesity classes on the severity and mortality of COVID-19 was unclear so far. In the present meta-analysis, we analyzed the relationship between morbid obesity and severity or mortality of COVID-19. The results show patients with morbid obesity have higher risk for the severe outcome of COVID-19. Different from patients with obesity, morbid obesity is linked with higher mortality. Thus, patients with morbid obesity should be given more attention, especially when they are infected with COVID-19. However, available evidence in this field is extremely limited. More studies should be conducted to explore the different impacts of obesity classes on the outcome of COVID-19.

Because the severe outcomes of COVID-19 are influenced by multiple factors (64), obesity might be one of the important influencing factors. Obesity could induce and aggravate the severe clinical manifestations of COVID-19 through influencing metabolism, inflammation, immune responses, and other pathways (65). At the same time, other factors, such as age, diabetes, hypertension, and cardiovascular diseases, can also cooperate with obesity to play a part in the development of COVID-19 (66). Thus, to discuss more influencing factors of severe COVID-19 is of great value.

In the present meta-analysis, we performed a subgroup analysis to discuss the heterogeneity of the included studies. Different regions were chosen as parameters to evaluate the heterogeneity. The results show significant decreased heterogeneity in the regions of Europe and North America in COVID-19 studies, indicating the existence of specific regional characteristics of COVID-19 (67). For the included studies on influenza, we did not find a valuable factor that could be used as a parameter to decrease the heterogeneity. Common parameters used for subgroup analysis, such as age and sex, are not suitable in the present studies. Because some studies did not provide enough information on these influencing factors, it is difficult to conduct subgroup analysis. Future studies should pay more attention to detecting more influencing factors on the severity and mortality of COVID-19.

Numerous studies have indicated the influenza vaccination could help to protect patients with obesity from the risk of influenza (68). However, its effect on COVID-19 is not clear so far, and available information is controversial. Because of the coming winter, it will be a great challenge for the global healthcare systems because the epidemic of influenza season is on the way. Due to the lack of a COVID-19 vaccine, the immunization for influenza might be helpful to prevent the epidemic wave of seasonal influenza and the co-circulation of both influenza and COVID-19 (69). A study from Zanettini et al. shows the influenza vaccination is helpful to reduce the mortality of COVID-19 in the elderly population (70). However, the effect of the influenza vaccination on other populations in preventing COVID-19 risk is quite limited and needs more evidence in this field (71). High influenza vaccine uptake rates in a well-matched season between the circulating influenza strains and the vaccine influenza strains could reduce the epidemiological noise of influenza during the COVID-19 epidemic. Thus, for patients with obesity, children, elders, pregnant women, and patients with chronic diseases, the influenza vaccination is highly advised.

### Strengths

To our knowledge, this is the first meta-analysis to compare the effects of obesity on the severity and mortality of influenza and COVID-19. In addition to a rigorous methodology to conduct the meta-analysis, we also applied subgroup analysis, evaluation of publication bias, and sensitivity analysis. To further explore the risk-increasing effect of obesity specifically, we also performed analysis comparing BMI classes with each other.

### Limitations

One significant limitation of the present study is that the majority of the included studies are retrospective studies. Because the pandemic of influenza and COVID-19 are not designed or planned, it is difficult to conduct the cohort study further. Besides this, the high heterogeneity of included studies does limit the ability to accurately estimate the size of the effects. Moreover, the BMI definition and the criteria for the admission to ICU might be different across different countries, which might influence the final results to some degree. For the assessment of publication bias, we tried to apply the Begg’s test. Sensitivity analysis was also performed. However, due to the limited number of included studies and high heterogeneity, no conclusive information was provided.

### Implications for Future Research

Based on available evidence, obesity or BMI should be considered as an important parameter for a COVID-19 risk assessment model. More studies in this field are encouraged to further examine whether obesity is an independent risk factor or predictor. Besides this, future studies should include detailed anthropometric parameters of patients with COVID-19. Moreover, basic research exploring the underlying mechanism of COVID-19 and obesity are urgently needed in the near future.

## Conclusion

Consistent with influenza, COVID-19 patients with obesity and morbid obesity present with a higher risk for severe outcome and admission to ICU. No correlation was found between obesity and mortality of COVID-19. Thus, obesity or BMI should be recommended as important parameters for COVID-19 risk assessment. Special monitoring and earlier treatment should be implemented in COVID-19 patients with elevated BMI. Furthermore, prevention of obesity and regular physical activity are important methods to fight against the COVID-19 pandemic.

## Data Availability Statement

The original contributions presented in the study are included in the article/**Supplementary Material**. Further inquiries can be directed to the corresponding authors.

## Author Contributions

XZ and GW designed the study. XZ and QH wrote the paper. XG and GH selected the paper. ZL and YL did the data extraction and analysis. All authors contributed to the article and approved the submitted version.

## Funding

This work was supported by grants (GW) from the National Natural Science Fund of China (81670732, 81970687) and Department of Science and Technology of Jilin Province(201909010006JC, 20170623005TC). This work was also supported by grants (XZ) from the National Natural Science Fund of China (81900726) and the 10th Youth Project of the First Hospital of Jilin University (JDYY102019025).

## Conflict of Interest

The authors declare that the research was conducted in the absence of any commercial or financial relationships that could be construed as a potential conflict of interest.
