# Supplementary material for: Obesity Increases the Severity and Mortality of Influenza and COVID-19: A Systematic Review and Meta-Analysis
Source: Front Endocrinol (Lausanne). 2020 Dec 21;11:595109. doi: 10.3389/fendo.2020.595109 (PMC7779975; doi:10.3389/fendo.2020.595109)

Supplementary Files

1. Subgroup analysis

1.1 subgroup analysis on the obesity and influenza infection.

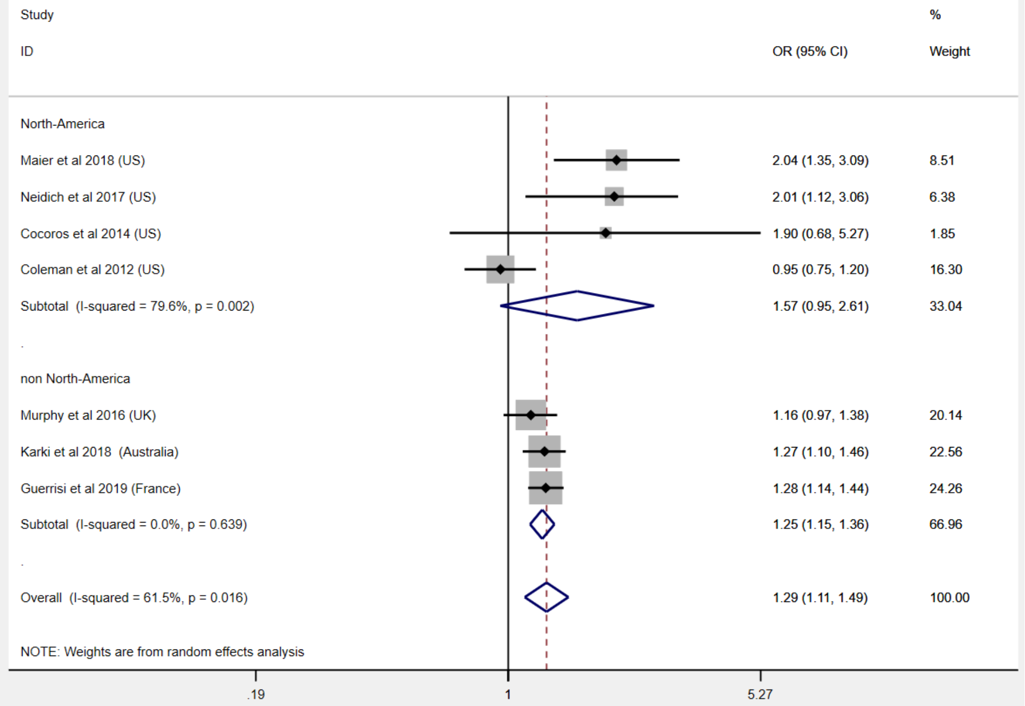

1.2 subgroup analysis on the obesity and influenza hospitalization

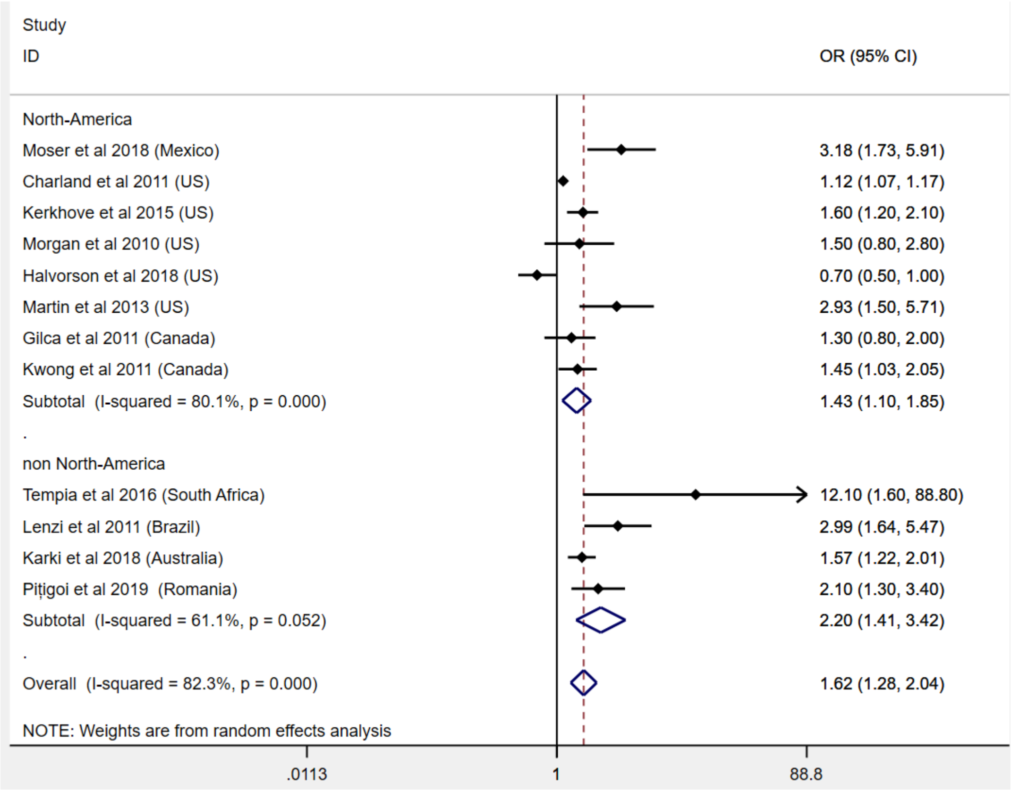

1.3 subgroup analysis on the obesity and severity of influenza and admission to ICU.

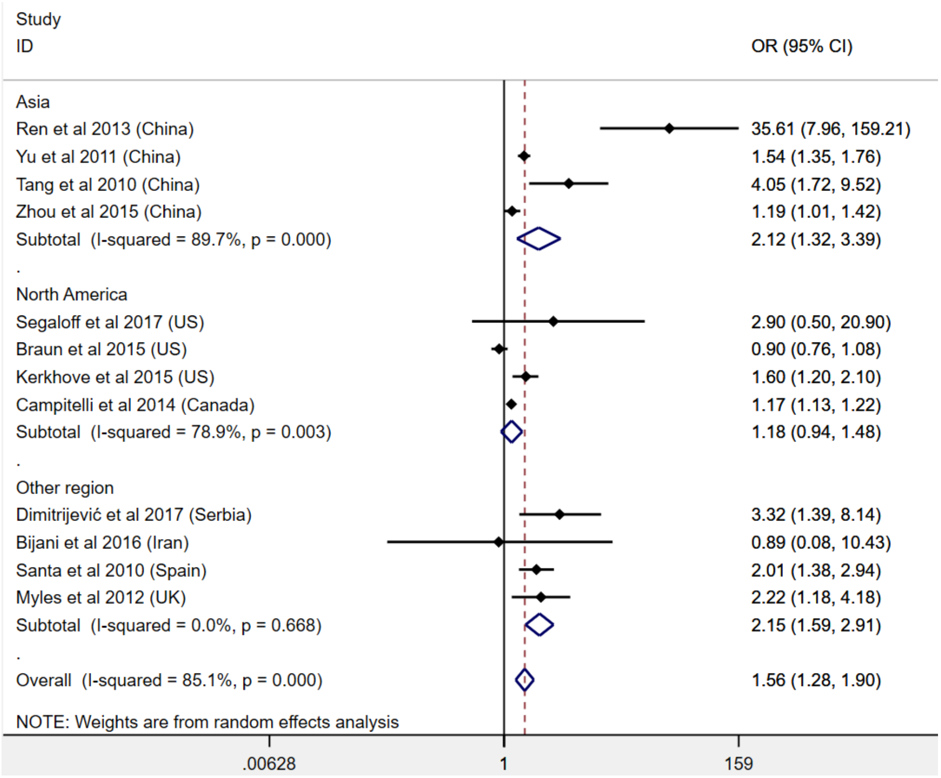

1.4 subgroup analysis on the obesity and mortality of influenza

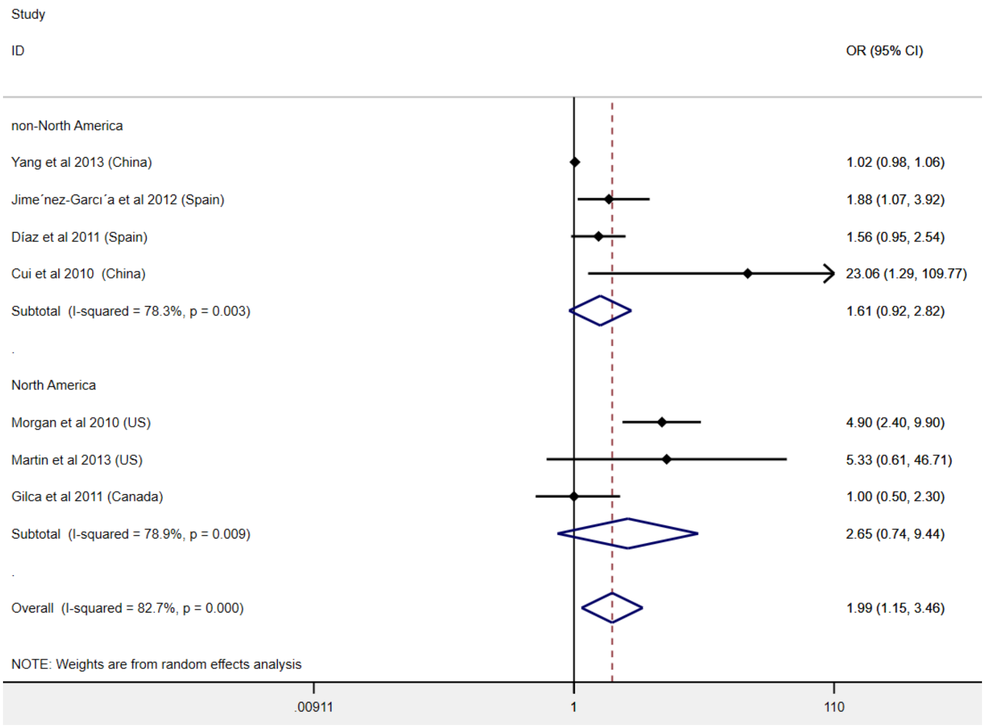

2. Publication bias assessment by Begg's test

Content	P value for Begg's test
Obesity and Influenza Risk	1.00
Obesity and Influenza Hospitalization	0.12
Obesity and severe outcome	0.174
Obesity and morality	0.533
Morbid obesity and severe outcome	1.00

3. Sensitivity analysis

3.1 Sensitivity analysis on the obesity and influenza risk

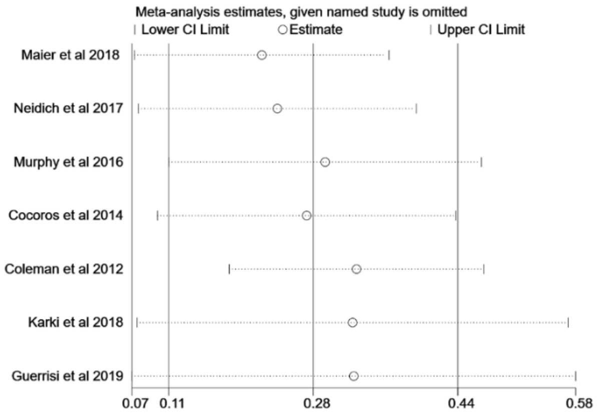

2.2 Sensitivity analysis on the obesity and influenza hospitalization

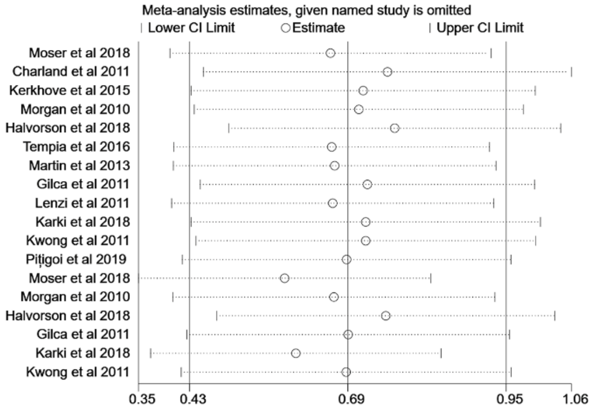

2.3 Sensitivity analysis on the obesity and influenza severity

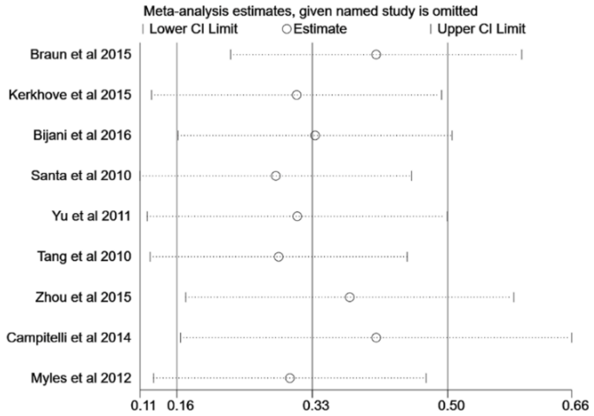

2.4 Sensitivity analysis on the obesity and COVID-19 severity

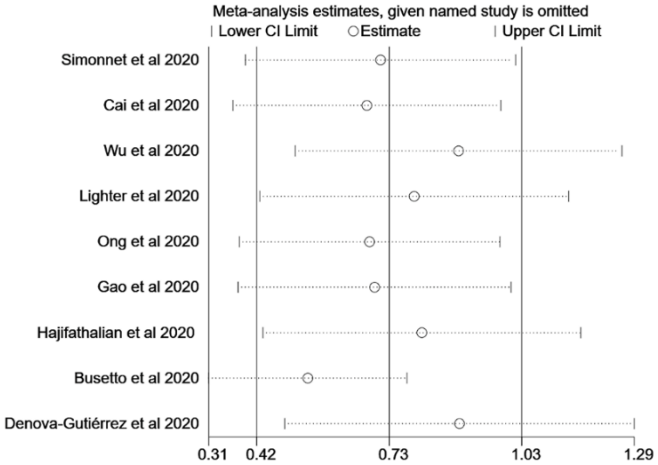

2.5 Sensitivity analysis on the obesity and COVID-19 death

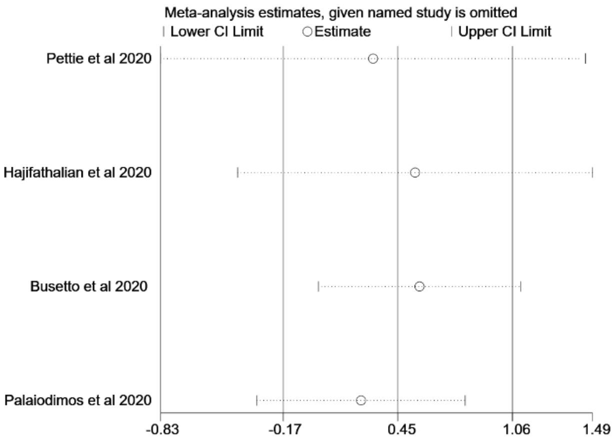

2.6 Sensitivity analysis on the obesity and influence death

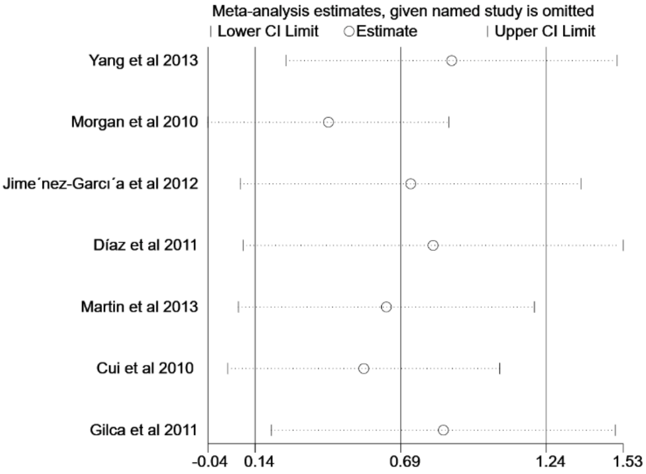

2.7 Sensitivity analysis on the morbid obesity and severity, mortality of influenza

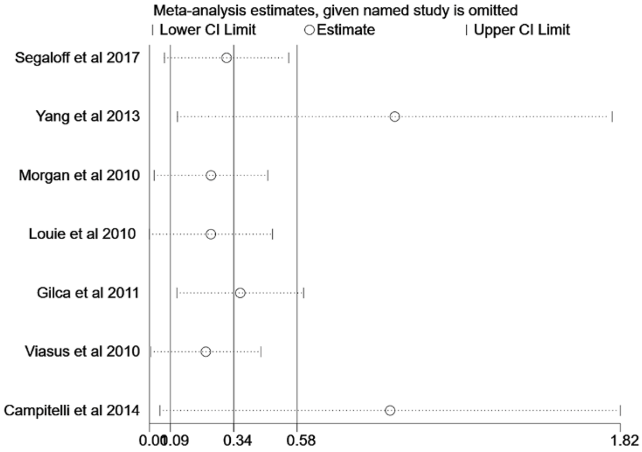

2.8 Sensitivity analysis on the morbid obesity and severity, mortality of COVID-19

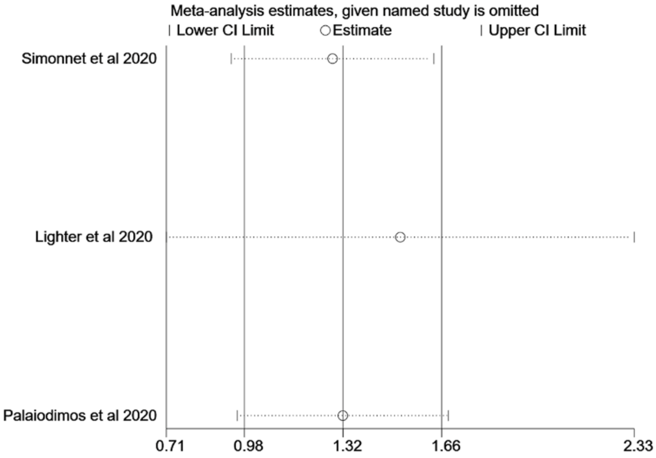

Supplement: Supplementary file 1 [file DataSheet_1.pdf]
